# In-gel fluorescence detection by DNA polymerase elongation

**DOI:** 10.1063/5.0021149

**Published:** 2020-11-20

**Authors:** Alden C. Moss, Amy E. Herr

**Affiliations:** 1 University of California, Berkeley/University of California, San Francisco Graduate Program in Bioengineering, Berkeley, California 94720, USA; 2 Department of Bioengineering, University of California, Berkeley, Berkeley, California 94720, USA

## Abstract

Fluorescence-based DNA readouts are increasingly important in biological research, owing to enhanced analytical sensitivity and multiplexing capability. In this study, we characterize an in-gel polymerase elongation process to understand the reaction kinetics and transport limitations, and we evaluate DNA sequence design to develop signal amplification strategies. Using fluorescently labeled nucleotides, we scrutinize polymerase elongation on single-stranded overhangs of DNA immobilized in polyacrylamide hydrogels. When polymerase elongation reactions were carried out with reactants diffused into the gels, we observed reaction completion after 2 h, indicating that the process was efficient but much slower than that predicted by models. Confocal microscopy revealed a nonuniform post-reaction fluorescence profile of the elongated DNA throughout the depth of the gel and that the time for complete fluorescence penetration was proportional to the immobilized DNA concentration. These observations suggest retarded diffusion of the polymerase, attributable to interactions between diffusing polymerase and immobilized DNA. This study will ultimately inform assay design by providing insight into the reaction completion time to ensure spatial uniformity of the fluorescence signal. In agreement with our hypothesis that incorporation of multiple labeled nucleotides per DNA strand results in an increased signal, incorporation of four labeled nucleotides resulted in a 2.3-fold increase in fluorescence intensity over one labeled nucleotide. Our results further suggest that the fluorescence signal increases with spacing between labeled nucleotides, validating the number of and spacing between labeled nucleotides as tunable parameters for signal amplification. In-gel polymerase-based fluorescence readout is promising for signal amplification when considering both transport limitations and DNA sequence design.

## I. INTRODUCTION

The advantageous physical, chemical, and optical properties of hydrogels make the substrate material well suited for a range of bioanalyses.^1–6^ Such assays often depend on fluorescence for the readout signals, and many rely on fluorescence-based DNA readouts. Fluorescence-based DNA readouts are advantageous because these readouts harness the power of DNA technologies, namely target multiplexing capability and signal amplification, while retaining spatial information and allowing for easy incorporation into existing fluorescence imaging workflows.^7–10^ *In situ* sequencing is an increasingly popular application of fluorescence-based DNA readouts, which provides a base-by-base readout of a DNA sequence while preserving spatial context.^7,9,10^

Either recent *in situ* sequencing studies have not been carried out in a hydrogel matrix, in the case of BaristaSeq,^9^ or signal amplification has been carried out before embedding the tissue in the gel matrix, as is the case in STARmap.^10^ A complete in-gel readout will be essential for some in-gel assays, such as single-cell immunoblotting, which involves gel electrophoretic separations of biological molecules prior to target detection.^4–6^ Additionally, the *in situ* sequencing methods mentioned above have utilized rolling circle amplification (RCA), which involves a ligation and an amplification step prior to fluorescence readout, resulting in a long assay with many steps. A readout with fewer steps and the use of one enzyme would be advantageous, resulting in a shorter assay timescale, less complexity, use of fewer enzymes, and less variability introduced from additional steps. An early approach to *in situ* sequencing of polymerase colonies (polonies) formed after in-gel polymerase chain reaction (PCR) involved only a single in-gel readout step based on polymerase elongation of single-stranded overhangs with labeled nucleotides but lacked signal amplification.^7^ A similar readout strategy was applied by Goltsev *et al.* to DNA oligomers conjugated to antibodies to achieve highly multiplexed immunohistochemical staining of mouse spleen tissue slices, but this was not an in-gel assay and again did not incorporate signal amplification.^8^ The potential exists for translating such a readout to in-gel nucleic acid and protein assays for ultrasensitive target detection. However, development and characterization of a completely in-gel assay readout that incorporates signal amplification will be necessary. A comparison of various *in situ* sequencing methods can be found in Table S1.

More broadly, thorough characterization of these polymerase elongation processes within hydrogels is lacking. Hydrogels introduce transport limitations since diffusion is hindered in the porous bulk of the material.^11,12^ Size exclusion-based thermodynamic partitioning, which limits the concentration of solute in the gel at equilibrium, is another factor in hydrogel-based assays.^13^ Mitra *et al.* evaluated *in situ* sequencing of polonies in a 40 *μ*m thick, 8% total acrylamide (8% T) polyacrylamide gel and observed an incomplete reaction after incubation times of up to 6 min.^7^ They concluded that the reaction was inefficient but did not characterize the process with regard to reaction kinetics or diffusive transport. Characterizing these aspects of polymerase elongation of DNA within hydrogel matrices would inform assay design.

Also important to the design of the fluorescence readout mechanism is the relationship between the fluorescence signal, number of incorporated labeled nucleotides per strand, and spacing between labeled nucleotides. In investigating readout of multiple base repeats, Mitra *et al.* observed attenuated fluorescence signals,^7^ which the authors attributed to self-quenching of the adjacent fluorophores. Relevant to mitigating signal attenuation, increased spacing between labeled nucleotides may be an option, when the sequence of interest is attached to a readout probe. Given that previous studies have scrutinized only a single labeled nucleotide incorporated per DNA strand, incorporation of multiple labeled nucleotides may also form the basis for design of signal amplification strategies.^8^ Such an approach would allow for a one-step reaction for integration of signal amplification with the polymerase elongation readout.^7,8^ Understanding the impact of DNA sequence design on the fluorescence signal generated by polymerase elongation with labeled nucleotides will ultimately inform assay design to maximize the fluorescence signal.

Studies of hydrogel-based assays have modeled the transport of reagents into a gel and the reaction kinetics in the gel to give assay design parameters such as reaction times and gel thicknesses.^11,14–16^ One particularly useful framework is the Damköhler number (Da), which describes the ratio of the characteristic transport time to the characteristic reaction time. Damköhler analysis has provided insight into the contributions of transport and reaction kinetics to the assay timescale and concentration profiles in the gel under different conditions.^11,14–16^ Specifically, modeling of the transport and reaction kinetics of in-gel polymerase elongation will provide an understanding of whether the process is limited by the transport of the polymerase into the gel or the polymerase elongation reaction of the immobilized DNA strands.

In this study, we develop design rules for polymerase elongation as a mechanism for fluorescence signal readout and amplification in hydrogel-based assays. To this end, we prepare polyacrylamide gels with DNA immobilized in the bulk to (i) characterize the effects of the reaction kinetics and transport properties on the in-gel polymerase elongation timescale and (ii) characterize the effects of DNA sequence design on the fluorescence signal. We scrutinize two hypotheses: first, that the timescale of polymerase elongation is limited by polymerase transport and reaction kinetics vs inherent reaction inefficiency. Second, that the fluorescence signal is modulated by both the number and spacing of incorporated labeled nucleotides per strand, which leads to a tunable approach to signal amplification.

## II. RESULTS AND DISCUSSION

### A. In-gel polymerase elongation model suggests a transport-limited process

**FIG. 1.**
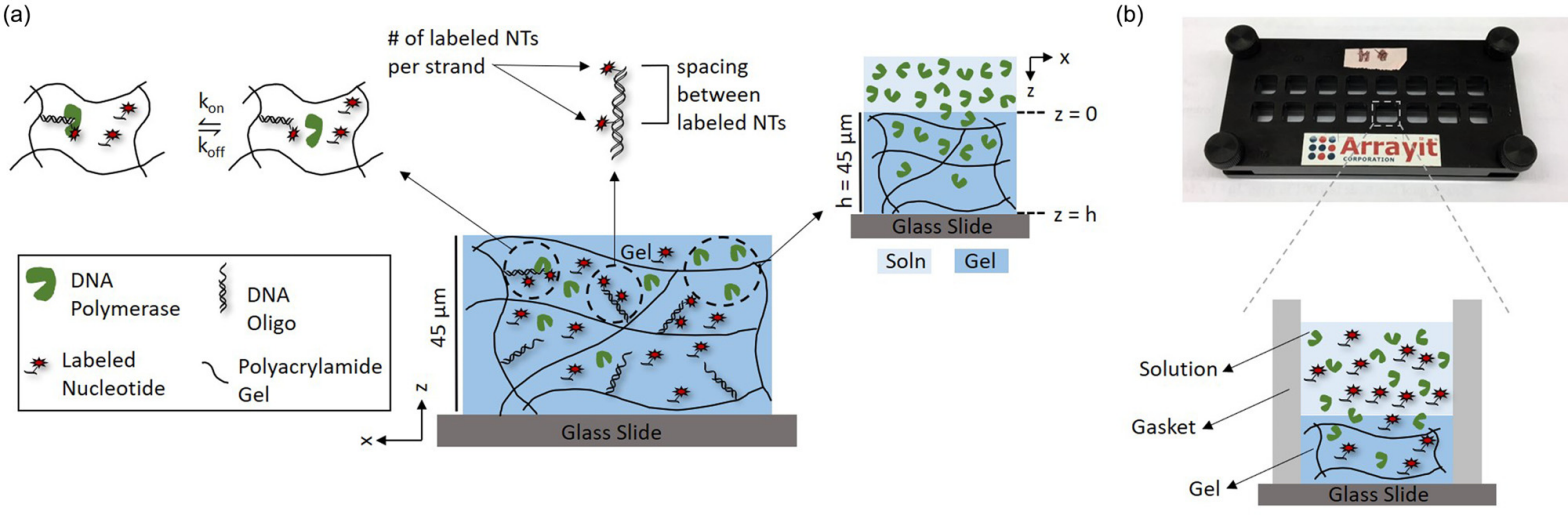
Polymerase reaction kinetics, DNA sequence design, and polymerase transport in the gel are the primary factors hypothesized to impact the fluorescence signal resulting from polymerase elongation in hydrogels. (a) We hypothesize (1) that the process is transport limited and (2) that signal amplification can be achieved by strategic DNA sequence design. The rate limiting step of the polymerase elongation reaction (schematic on the left) is the detachment of the polymerase from an elongated strand of DNA, which gives a characteristic time of 1.0 s. The characteristic transport time for the polymerase through a 45 *μ*m thick gel (schematic on the right) is 74 s, assuming a modified Stokes–Einstein diffusion coefficient that takes into account hindered diffusion in the gel pores, suggesting a transport limited process. DNA sequence designs that increase the number of labeled nucleotides incorporated per strand and the spacing between labeled nucleotides (schematic in center) are hypothesized to increase the fluorescence signal. (b) The device consists of a gel bonded to a glass slide and mounted in a microarray cassette. Gel regions are separated by a gasket. Reagent solutions are applied to the top of each gel region and diffuse into the gel.

Our model system consists of a polyacrylamide gel copolymerized with 5′ acrydite-terminated DNA oligomers. Single-stranded overhangs are generated by hybridizing a longer strand to the immobilized DNA. Polymerase elongation solution that contains Klenow (exo-) polymerase and Alexa Fluor 647-labeled dUTP is then introduced to the surface of the gel, allowing the reagents to diffuse into the gel in the z-direction and elongate the single-stranded overhangs. We assume that the process will not be limited by diffusion of the dNTPs due to their small size^17^ and that depletion zones will not form due to the large excess of fluid volume above each gel region. Therefore, the two primary factors governing this process are the polymerase transport into the gel and the polymerase reaction kinetics, as illustrated in Fig. 1. In considering both polymerase transport into the gel matrix and polymerase reaction kinetics, we used a Damköhler number (Da) to delineate transport and reaction limited regimes for the system. Da is the ratio of the characteristic transport time to the characteristic reaction time, such that Da ≫ 1 indicates a transport limited process, while Da ≪ 1 indicates a reaction limited process.^14^ Previous studies have utilized Da analysis as an assay design framework to achieve a maximized, uniform fluorescence signal and a minimized assay time.^15,16^ Da ≫ 1 in our system represents a case where the polymerase is diffusing into the gel much slower than the immobilized DNA that is elongated, while Da ≪ 1 indicates that the polymerase is diffusing throughout the depth of the gel at a much faster rate than the elongation reaction is occurring. Understanding the regime in which the in-gel polymerase elongation process operates will inform optimization of assay design parameters, including the incubation time and gel thickness, for a complete and uniform reaction throughout the depth of the gel.

In our model, polymerase transport in the gel was represented with Fickian diffusion in one dimension [Eq. (1)] and a modified Stokes–Einstein diffusivity with a correction that accounts for hindered diffusion within the gel matrix [Eq. (2)].^11,12,18–20^ A characteristic transport time can then be calculated with Eq. (3),

$$\frac{\partial C}{\partial t}=D_{\mathrm{gel}}\frac{\partial^{2}C}{\partial z^{2}},$$ (1)

$$D_{\mathrm{gel}}=D_{\mathrm{soln}}e^{-K_{c}R_{h}\varphi^{0.75}},$$ (2)

$$\tau_{\mathrm{transport}}=\frac{h^{2}}{2D_{\mathrm{gel}}},$$ (3)

where C is the concentration of the polymerase (M), D_gel_ is the diffusion coefficient in the gel (m^2^/s), D_soln_ is the diffusion coefficient in the solution calculated with the Stokes–Einstein equation (m^2^/s), K_c_ is a proportionality constant (m^−1^), R_h_ is the hydrodynamic radius of the species diffusing (m), φ is the polymer volume fraction of the gel (dimensionless), and h is the gel height (m). All values included in the models are tabulated in Table S2. Constant source diffusion in the z-direction was assumed with a zero-flux boundary at the gel–glass interface.

We also constructed a model [Eq. (4)] for the polymerase reaction kinetics based on the literature,^21^ which suggests that the rate-limiting step for Klenow (exo-) polymerase is the detachment of the polymerase from the elongated DNA strand (Fig. 1). A resulting characteristic reaction time based on this model can then be calculated using Eq. (5). For the calculation of the characteristic reaction time, the in-gel enzyme concentration was estimated based on the Ogston model of size-exclusion based thermodynamic partitioning, which yielded a partition coefficient of 0.46 for Klenow (exo-) polymerase in an 8% T gel,^13^

$$DE\overset{k_{\text{on}}}{\underset{k_{\text{off}}}{⇋}}D+E,$$ (4)

$$\tau_{\mathrm{rxn}}=\frac{1}{k_{\mathrm{off}}+K\left[E\right]k_{on}},$$ (5)

where D is the concentration of elongated DNA strand immobilized in the gel (M), E is the concentration of the polymerase enzyme (M), K is the partition coefficient (dimensionless), k_on_ (M^−1^ s^−1^) is the kinetic rate constant of binding, and k_off_ (s^−1^) is the kinetic rate constant of dissociation of the polymerase from the elongated strand.

**FIG. 2.**
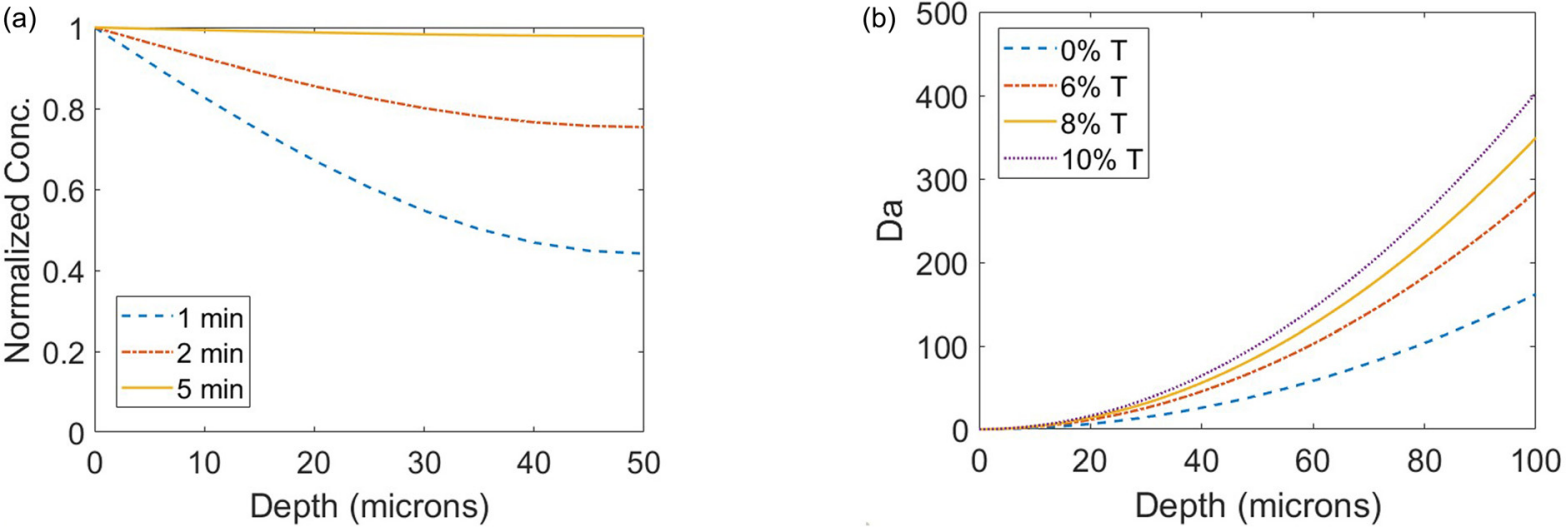
Models of in-gel polymerase diffusion and reaction indicate that the process is transport limited across conditions. (a) Transient 1D model of constant source diffusion of Klenow (exo-) DNA polymerase indicates that a uniform concentration profile in the z-direction is reached after about 5 min and (b) the diffusion and polymerase elongation reaction models predict Da > 1 across a wide range of gel thicknesses and all gel densities, including free solution (0% T), which suggests that the process is transport limited across the vast majority of conditions.

These models predict a characteristic transport time of 74 s and a characteristic reaction time of 1.0 s in our system, which consists of a 68 kDa Klenow (exo-) polymerase diffusing through a 45 *μ*m thick 8% T gel. The resulting value of Da is ∼70, which indicates that the system is transport limited. For comparison, the resulting value of Da for the dNTPs is ∼3, which suggests a transport timescale on the same order of magnitude as the reaction timescale. The Da regime estimate suggests that polymerase transport is the limiting factor. Given the expected transport limited regime of this system, we estimated that diffusion of the polymerase throughout the depth of the gel (in the z-direction) occurs within 5 min [Fig. 2(a)]. More broadly speaking, Da analysis indicates that the process is largely transport limited even in solution, indicating that the process is transport limited across different gel densities with Da scaling with the square of the gel thickness [Fig. 2(b)].

### B. In-gel polymerase elongation kinetics are slower than that predicted by reaction and transport limitations

**FIG. 3.**
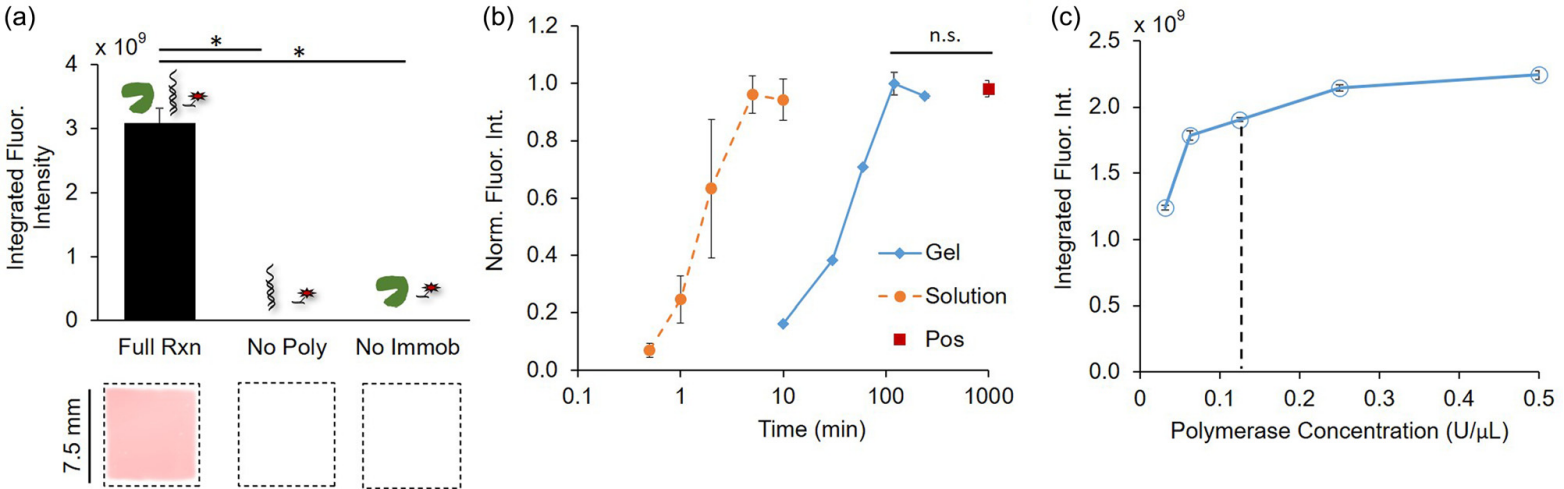
In-gel polymerase elongation generates a strong signal with minimal background and reaches completion after a 2 h incubation at room temperature. (a) Integrated fluorescence intensity and corresponding micrographs from the complete in-gel reaction demonstrate a strong fluorescence signal, while minimal fluorescence was observed in the negative controls that omitted polymerase (No Poly) or immobilized DNA (No Immob) (n = 5), (b) normalized integrated fluorescence intensity of gels exposed to polymerase elongation solution demonstrates complete elongation after 2 h, as indicated by a positive control of a hybridized labeled complementary strand (Pos), while completion is achieved in ≤5 min in solution (n = 3, normalization to max intensity of each condition), and (c) in-gel experiments with an increased concentration of polymerase above 0.125 U/*μ*l used in most experiments (indicated by the dotted line) resulted in a minimal increase in fluorescence for a 30 min incubation (n = 3). Statistical significance was determined by pairwise Mann–Whitney U-tests, ^*^p < 0.05, p ≫ 0.05 for n.s., and standard deviation error bars are shown.

Next, we measured the timescale of in-gel polymerase elongation. First, we sought to evaluate the background signal attributable to retention of fluorescently labeled nucleotides within the gel during the polymerase elongation process. As retention of fluorescent probes is a direct contributor to in-gel assay background and noise, it is important to understand how much additional background in the gel arises due to the introduction of fluorescently labeled nucleotides.^13,14^ The fluorescence signal was evaluated by exposing regions of gel that contained immobilized DNA to a polymerase elongation solution that contained Klenow (exo-) polymerase and dNTPs (including AF 647-labeled dUTP substituted for dTTP). The background signal arising from retention of labeled nucleotides was estimated using negative controls that contained the labeled nucleotides but omitted either the polymerase or the immobilized DNA. The negative controls exhibited fluorescence signal that was three orders of magnitude lower than the full reaction condition, indicating minimal background from retained fluorescently labeled nucleotides [Fig. 3(a)]. The strong fluorescence signal and minimal background suggest that polymerase elongation with fluorescently labeled nucleotides is an effective strategy for DNA readout in polyacrylamide gels.

Second, we evaluated how the kinetics of the in-gel polymerase elongation process differ from in-solution kinetics. A previous study evaluated polymerase reaction kinetics on a surface with immobilized DNA, another assay format that employs immobilized DNA, and found that the polymerase reaction kinetics were comparable to those in solution.^22^ Additionally, the literature suggests that hydrogels are advantageous environments for reactions—as compared to surfaces—due to a larger number of possible accessible molecular orientations.^23,24^ Therefore, we predict that the in-gel polymerase reaction kinetics will occur at the same rate as in solution. The hypothesis of comparable in-gel reaction kinetics is supported by in-gel PCR studies that employed standard PCR protocols without any need for increasing the times of the elongation steps.^25,26^ However, as compared to this study, the previous in-gel PCR studies diffused all reactants into the gel (rather than immobilized), employed higher temperatures, and used different polymerases. In the most direct comparison to our study, where immobilized DNA was isothermally elongated with fluorescently labeled nucleotides in a 40 *μ*m thick 8% T gel, Mitra *et al.* found that the reaction was not complete within the expected timescale of <6 min.^7^ They attributed the incomplete reaction to inefficient incorporation of the nucleotides but did not run the reaction to completion. Our transport model indicates that the time necessary for diffusion of the polymerase into the gel is on the same order of magnitude as even the longest reaction times employed by Mitra *et al.* Thus, we hypothesize that the reaction was not run to completion due to the transport limitations in the gel. As the polymerase is often at a low concentration in PCR and, thus, the rate-limiting species, increasing the polymerase concentration has been shown to result in a linear increase in the fluorescence for a given time point.^27^ Therefore, modulating the polymerase concentration will allow us to evaluate whether reaction limitations are dominant in the system.

While polymerase elongation in solution reached completion after 5 min, the fluorescence signal of the in-gel reaction increased for room-temperature incubation times up to 2 h, at which point the fluorescence plateaued [Fig. 3(b)]. No statistically significant differences (Mann–Whitney U-tests, p ≫ 0.05) in the normalized fluorescence intensity were observed between the 2 h timepoint [1.0 ± 0.03 relative fluorescence units (RFU)], the 4 h timepoint (0.96 ± 0.03 RFU), and the positive control (0.98 ± 0.03 RFU), in which a fluorescently labeled complementary strand was hybridized to the immobilized DNA in the gel. Comparable signals between the fluorescence plateau values and the positive control indicate that all of the immobilized DNA in the gel is elongated by the polymerase after long incubation times. The observed discrepancy between in-gel and in-solution kinetics could be attributed to either reaction limitations or transport limitations resulting from diffusion of the polymerase. To evaluate whether reaction limitations are dominant, we varied the concentration of the polymerase, the rate-limiting species. Increasing the concentration of the polymerase from 0.125 U/*μ*l to 0.5 U/*μ*l resulted in an 18% increase in the fluorescence signal after a 30-min incubation at room temperature [Fig. 3(c)]. The resultant 18% increase in fluorescence from quadrupling the polymerase concentration is minimal compared to the expected linear relationship between the polymerase concentration and reaction progress based on the literature.^27^ Taken together, the results presented here indicate that the reaction is efficient, as all immobilized DNA in the gel is elongated upon reaction completion, and that the discrepancy between the in-gel and in-solution kinetics is likely not due to reaction limitations. Therefore, we posit that transport limitations are responsible for the discrepancy between the in-gel and in-solution kinetics of the polymerase elongation process.

### C. Confocal microscopy reveals a nonuniform fluorescence profile throughout the depth of the gel

**FIG. 4.**
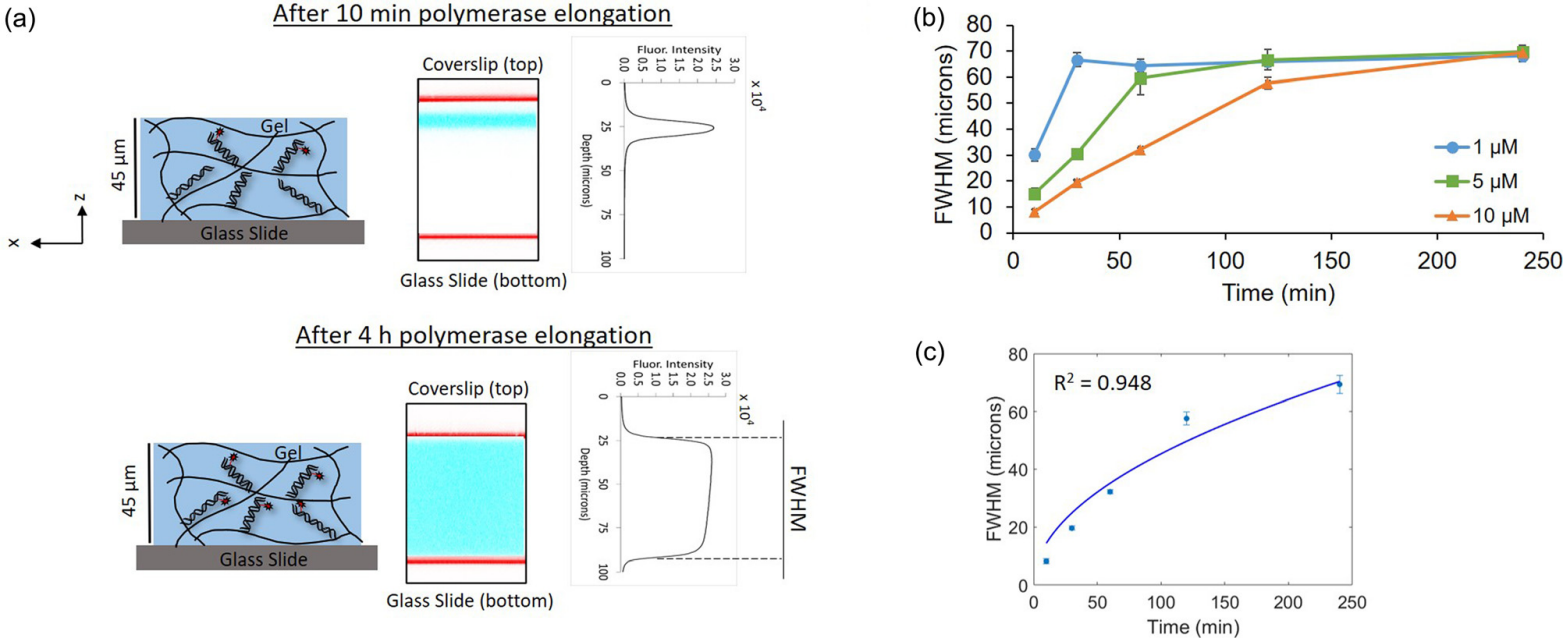
Confocal analysis suggests that the in-gel polymerase elongation process is transport limited and supports retarded diffusion. (a) Fluorescence micrographs demonstrate increased fluorescence penetration after a 4 h incubation with the polymerase elongation solution (polymerase + labeled dUTP, washed before imaging) than after a 10 min incubation, with similar maximum fluorescence intensity values in the brightest portions of the gel (red = glass, blue = gel fluorescence, and white space between the coverslip and gel at 10 min due to a fluid layer). (b) Line plots of the full width at half maximum (FWHM) fluorescence within the gels for various concentrations of immobilized DNA indicate that the time for complete fluorescence penetration throughout the gel is proportional to the concentration of immobilized DNA (n = 3, standard deviation error bars). (c) A good fit (R^2^ = 0.948) was obtained when the FWHM data for the 10 *μ*M immobilized DNA condition were fitted to a mean square displacement (MSD) function, yielding an effective diffusion coefficient of 0.172 ± 0.06 *μ*m/s (95% C.I.).

To investigate the hypothesis of transport limitations contributing to the slow in-gel polymerase elongation process, we sought to measure the post-reaction fluorescence profiles throughout the depth of the gels. A high Da indicates that diffusion is occurring slower than the reaction that can take place, which results in a nonuniform concentration profile throughout a gel.^16^ Based on Da analysis of our system and the result of the minimal signal increase from increasing the polymerase concentration, we hypothesize that we will observe increased fluorescence penetration into the gel with an increased incubation time. Confocal microscopy with reflection images of the glass coverslip and glass slide to delineate the gel bounds allowed us to observe how deep the fluorescence penetrated into the gel at different time points [Fig. 4(a)]. Fluorescence that reaches from the glass coverslip on top of the gel to the glass slide on the bottom was observed at the 4 h time point, while fluorescence was observed through less than a quarter of the gel at the 10 min time point. While there was a substantial difference in the fluorescence penetration, the maximum fluorescence intensity in the brightest portions of the gel is similar in both the 10 min and 4 h time points at 2.51 ± 0.16 and 2.54 ± 0.06 × 10^4^ arbitrary fluorescence units (AFUs), respectively (n = 3). This result is consistent with transport limitations rather than reaction limitations. In the case of a reaction limited system with a low value of Da, we would have expected to see an increase in the maximum fluorescence intensity rather than the fluorescence penetration since diffusion of the polymerase would be occurring at a much faster rate than the reaction. While the theoretical analysis indicated a transport limited process, the extent of this limitation turned out to be much greater than predicted. Our diffusion model predicted that the polymerase would diffuse through the thickness of the gel within 5 min, which is much less than the 2–4 h necessary for complete fluorescence penetration. This discrepancy suggests additional contributing factors, such as retarded diffusion. Retarded diffusion is a phenomenon that has been observed when DNA oligomers are hybridized to single-stranded DNA immobilized in a gel and occurs as a result of interactions between the DNA strands that hinder the diffusion through the thickness of the gel.^28^ The amount of retardation is proportional to the concentration of immobilized DNA in the gel and the affinity between the interacting species, and the resulting rate of diffusion can be several orders of magnitude slower than simple diffusion.^28^ While retarded diffusion of enzymes in this manner has not been previously described, polymerase binding of a DNA template occurs with similar high affinity to that of DNA hybridization.^21^ Thus, it is possible that an analogous mechanism of retarded diffusion of the polymerase results in the slow in-gel elongation kinetics that we have observed.

To further evaluate our hypothesis of retarded diffusion, in-gel polymerase elongation was evaluated with different concentrations of immobilized DNA, as the characteristic diffusion time in retarded diffusion is proportional to the concentration of immobilized DNA. Polyacrylamide gels with 1 *μ*M and 5 *μ*M immobilized DNA were evaluated, in addition to the gels with 10 *μ*M immobilized DNA that were evaluated in all other experiments. We hypothesized that the time for complete fluorescence penetration throughout the depth of the gel would be dependent on the concentration of immobilized DNA in the gel. Fluorescence intensity profiles in the z-direction were again measured by confocal microscopy with the length in the z-direction that exhibited >50% of the max fluorescence intensity (full width at half maximum, FWHM) designated as a quantitative metric for the depth of fluorescence penetration in the gel. The results suggest that complete polymerase elongation throughout the depth of the gel, indicated by a plateau FWHM value of ∼65–70 *μ*m, was achieved in under 30 min for 1 *μ*M immobilized DNA, 1 h for 5 *μ*M immobilized DNA, and under 4 h for 10 *μ*M immobilized DNA [Fig. 4(b)]. Plateau times were determined by a Kruskal–Wallis test, which resulted in p > 0.05 when the 30 min, 1 h, and 2 h time points were grouped together for the 1 *μ*M condition and when the 1 h, 2 h, and 4 h time points were grouped together for the 5 *μ*M condition. These reaction completion times suggest that the time for completion of in-gel polymerase elongation is proportional to the concentration of immobilized DNA, which is consistent with our hypothesis of retarded diffusion. Further support for the retarded diffusion hypothesis was obtained by fitting a 1D diffusion model based on mean square displacement (MSD) [Eq. (6)] to the FWHM data from the 10 *μ*M immobilized DNA condition. There is a literature precedent for this type of fitting strategy, where the position of the diffusion front was represented by the distance corresponding to half the maximum signal and fitted to a mean square displacement model.^29^ Only the 10 *μ*M data were fitted due to the lack of data points for a complete curve in the other conditions. The model was a good fit (R^2^ = 0.948), with a fitted effective diffusion coefficient of 0.172 ± 0.06 *μ*m/s [Fig. 4(c), 95% C.I.]. When compared to the predicted in-gel diffusion coefficient for simple diffusion, the fitted effective diffusion coefficient was 80-fold smaller. In retarded diffusion, the effective diffusion coefficient can be related to the simple diffusion coefficient by the retardation coefficient K [Eq. (7)], where K is the product of the association constant of the interacting species (equivalent to k_on_/k_off_) and m, the concentration of immobilized DNA [Eq. (8)],^28^

$$z=\sqrt{2D_{\mathrm{eff}}t},$$ (6)

$$D_{\mathrm{eff}}=\frac{D_{\mathrm{gel}}}{K},$$ (7)

$$K=\frac{k_{on}}{k_{\mathrm{off}}}m.$$ (8)

Based on the retarded diffusion equations and the model fit, the retardation coefficient for our system is K = 80. Estimation of K based on the association constant from the literature^21^ yields a value of K = 2000, which is higher than our estimated value. This discrepancy could be explained by the difference between DNA hybridization, which the retarded diffusion model is based on, and polymerase elongation. Polymerase elongation is a more complex process that involves association, extension of the DNA strand, and dissociation. Therefore, it is likely that modifications to the retarded diffusion model are necessary and a topic for future investigation. Overall, the dependence of the fluorescence penetration depth on the DNA concentration and the good fit to a diffusion model support a retarded diffusion-type mechanism.

While our study specifically focused on thin, dense gels with immobilized DNA concentrations of ≥1 *μ*M, these findings likely apply to many other hydrogel-based systems, such as gel cleared tissue. Gel-cleared tissue studies employ gels that are less dense than the gels used in our study but are often 1–2 orders of magnitude thicker.^1^ Additionally, specific intracellularly expressed proteins are often immobilized in hydrogels at concentrations on the order of 0.1–1.0 *μ*M,^4^ the upper end of which, after binding with DNA-labeled antibodies, would be close to what was investigated in our study. Since our Damköhler analysis indicates that even less dense gels are highly transport limited (Fig. 2), the slow transport phenomenon described here could be observed in a 1-mm thick 4% T gel with 0.5 *μ*M of a specific protein, a realistic scenario for gel-cleared tissue.^1–3^ The broader implication is that an understanding of slow transport through thick matrices containing immobilized species will provide assay design parameters including concentrations, timescales, and methods of introducing reagents. Our findings suggest that an understanding of the local in-gel concentrations of an immobilized species could be important, as the variable local concentration in a gel has the potential to introduce spatial nonuniformity, particularly in the z-direction. Therefore, running the reaction to completion will be essential to ensure spatial uniformity. As the slow transport of the polymerase results in a slow, cumbersome assay, in-gel polymerase elongation could benefit from the development of strategies that reduce the assay time. Mitra *et al.* developed a strategy for faster completion of the process where the polymerase was incorporated in a denser gel precursor that was poured on top of the primary gel and polymerized before adding other reaction components.^7^ Building on the results presented in our study has the potential to further advance strategies that improve transport in hydrogel matrices. These results also suggest that slower than expected transport may occur in other hydrogel systems that involve reactions and/or affinity-based interactions. In conclusion, further study will be necessary to understand these transport phenomena in hydrogels and develop assay design rules for applications that rely on thick gels, dense gels, and gels with high concentrations of immobilized DNA.

### D. Increased number of and spacing between labeled nucleotides result in an increased fluorescence signal

The second goal of this work was to evaluate various DNA sequence designs that incorporate multiple labeled nucleotides to determine the effect on the fluorescence signal, as one labeled nucleotide incorporated per strand has been the focus of previous studies.^7,8^ Ultimately, we sought to develop design rules for signal amplification via in-gel polymerase elongation and improve the reading out of repeat bases. Two key sequence design parameters were evaluated: the number of labeled nucleotides incorporated per strand and the spacing of unlabeled nucleotides between each labeled nucleotide. To the best of our knowledge, no other studies have evaluated the effect of varying a specified number of labeled nucleotides incorporated into a DNA strand via polymerase elongation in a hydrogel matrix. A previous in-solution study evaluated chemically labeled DNA strands and found that for strands of a similar length, up to five Cy3 labels per strand resulted in increased fluorescence.^30^ Additionally, previous in-solution studies have successfully incorporated multiple labeled nucleotides per strand via polymerase elongation, but did not thoroughly characterize the fluorescence increase that results from a specified increase in the number of labeled nucleotides incorporated.^31,32^ As the in-gel molecular accessibility of DNA is thought to be comparable to that in solution,^23,24^ we hypothesized that the relationship between the in-gel fluorescence increase and the number of incorporated labeled nucleotides would be similar to that in in-solution studies. Other key sequence design factors that we will be evaluating include the spacing between labeled nucleotides and the distance of labeled nucleotide incorporation sites from the end of the DNA strand. When fluorophores are <10 nm apart, self-quenching can occur due to homo-Förster resonance energy transfer (FRET).^33,34^ Additionally, formation of truncation products during polymerase elongation with labeled nucleotides has been observed when the labeled nucleotide is incorporated close to the end of the strand.^31,32^ Thus, we hypothesized that increasing the spacing between labeled nucleotides while being cognizant of the distance from the end of the strand will result in an increased fluorescence signal.

**FIG. 5.**
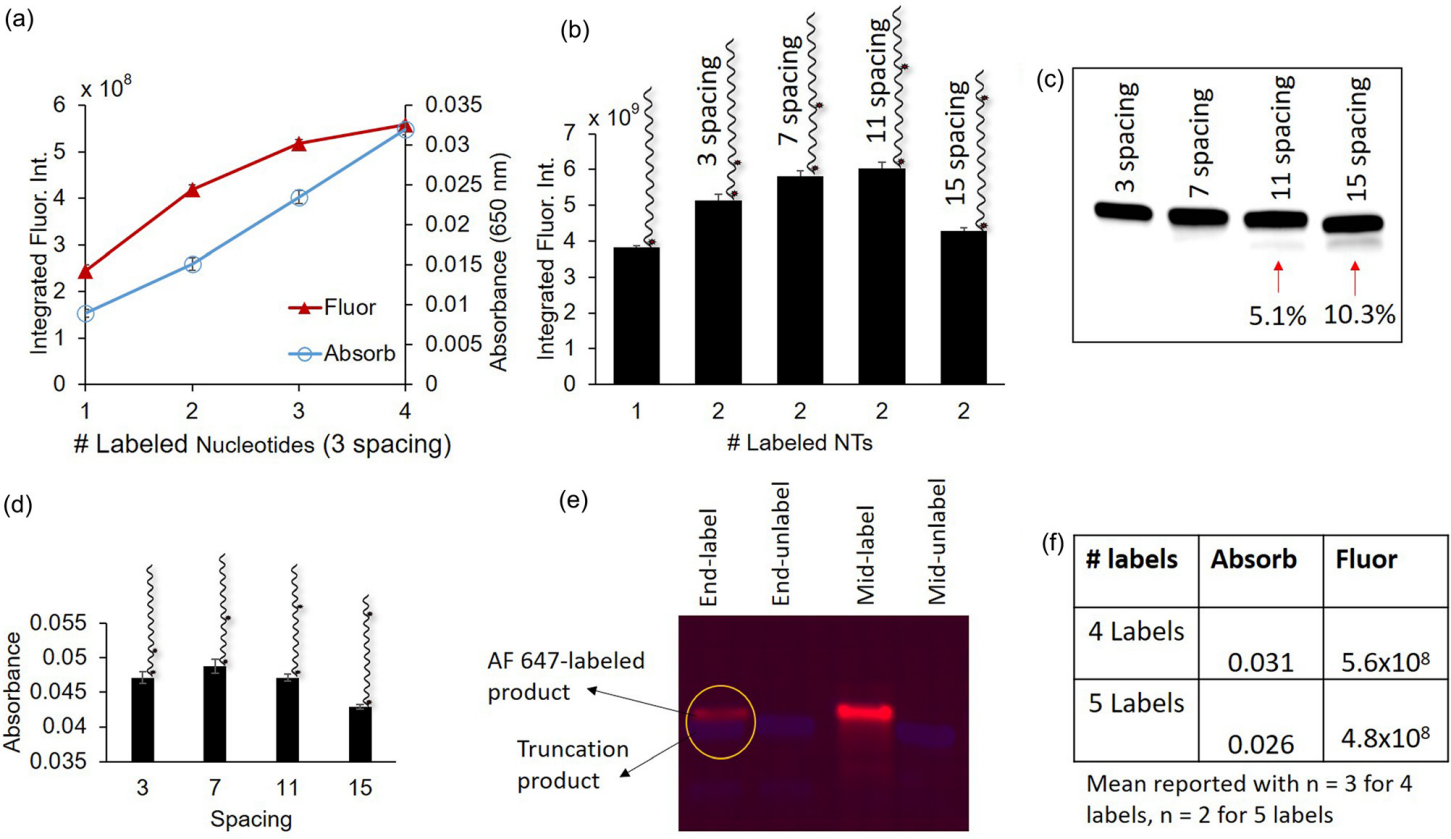
Fluorescence signal can be increased through incorporation of multiple labeled nucleotides and increased spacing between labeled nucleotides. (a) In-gel fluorescence intensity increases at a decreasing rate as multiple labeled nucleotides are incorporated, while the absorbance increase at 650 nm indicates complete incorporation of the labeled nucleotides, (b) DNA sequences with increased spacing between labeled nucleotides (representative schematic above each bar on the plot) result in increased in-gel fluorescence (sp indicates the spacing), and (c) sequences with varying spacings between labeled nucleotide incorporations were polymerase elongated in solution and run on a 15% TBE-urea gel, revealing lower molecular weight bands (indicated with arrows) below the primary band for the sequences with 11 and 15 spacings that comprise 5.1% and 10.3% of the total signal, respectively. This indicates the formation of truncation products that contain only one labeled nucleotide. (d) The absorbance after polymerase elongation in gel decreases for spacings greater than seven, which is indicative of polymerase truncation, (e) Polymerase truncation is observed when the labeled nucleotide is incorporated at the end of a strand, as indicated by the additional bands on the 15% TBE-urea gel (red is AF 647 from labeled nucleotides, blue is SYBR gold stain, label = AF 647-dUTP, and unlabel = dUTP), (f) a decrease in fluorescence and absorbance was observed when increasing the number of labeled nucleotides from 4 to 5 with three spacing between. n = 3 replicates with standard deviation error bars unless otherwise specified.

We first evaluated DNA sequence designs (Table S3) that could incorporate multiple labeled nucleotides with a spacing of three unlabeled nucleotides between each labeled nucleotide. Sequences that allowed for the incorporation of multiple labeled nucleotides resulted in increased fluorescence for up to four incorporated labeled nucleotides [Fig. 5(a)]. However, the increase tapered off for increasing numbers of labeled nucleotides such that a 2.3-fold signal increase was observed in a strand that incorporated four labeled nucleotides when compared to a strand that incorporated one labeled nucleotide. We did not observe this tapering off in the absorbance reading for each additional incorporated labeled nucleotide, indicating that the polymerase was incorporating the predicted number of labeled nucleotides into each sequence [Fig. 5(a)]. Interestingly, the fluorescence and absorbance decreased 15% and 18%, respectively, when the number of incorporated labeled nucleotides increased from 4 to 5 [Fig. 5(f)]. The decrease in fluorescence and absorbance is hypothesized to be due to incomplete incorporation of the labeled nucleotides that results from premature dissociation of the polymerase, resulting in truncation products. In agreement with other studies,^30–32^ we have observed in solution that DNA sequences in which the labeled nucleotides are incorporated at the end of the strand result in truncation products [Fig. 5(e)]. Additionally, our results closely match a previous study, in which the increase in the fluorescence signal was observed for up to five labeled nucleotides per strand.^30^ The tapering off of the fluorescence signal is likely a result of self-quenching since the spacing of three nucleotides between each labeled nucleotide corresponds to ∼1 nm (assuming 0.34 nm/nucleotide).^35^

To test our hypothesis of self-quenching, we investigated the effect of increased spacing between labeled nucleotides on the gel fluorescence. Increased spacing was hypothesized to mitigate self-quenching and result in increased fluorescence. When the spacing between two labeled nucleotide incorporation sites was increased from 3 to 7 unlabeled nucleotides, a 13% increase in the fluorescence signal was observed, and when increased from 3 to 11 unlabeled nucleotides, a 17% increase was observed [Fig. 5(b)]. When the spacing was increased from 11 to 15, a decrease in fluorescence was observed. A reaction in solution followed by separation on a 15% Tris-borate-EDTA (TBE)-urea gel revealed a truncation product for the 11 and 15 spacing sequences that was especially prevalent with the 15 spacing sequence, constituting 10% of the signal in that lane of the gel [Fig. 5(c)]. The truncation hypothesis was corroborated by absorbance readings of the gels that, when compared to the seven spacing case, revealed a 12% decrease in the absorbance for 15 spacing and a 3.4% decrease for 11 spacing [Fig. 5(d)]. We hypothesize that the formation of a truncation product is due to the second labeled nucleotide being incorporated closer to the end of the strand in the 11 and 15 spacing cases (Table S3). While increased spacing resulted in an increased fluorescence signal, even the signal corresponding to the 11 nucleotide spacing was still far from double that of one incorporated labeled nucleotide. Additionally, the fluorescence increase from 7 to 11 spacing was minimal at 3.8%, indicating diminishing returns for longer spacers. Diminishing returns in additional fluorescence for longer spacers are consistent with the theory on self-quenching because homo-FRET decreases exponentially with the increasing distance between fluorophores.^33^ Considering that the DNA strand length increases 0.34 nm per additional base and homo-FRET occurs when fluorophores are less than 10 nm apart, we hypothesize that a strand length of up to ∼30 bases may be necessary to completely eliminate self-quenching.^33,35^ Overall, our results support the hypothesis that self-quenching due to homo-FRET results in reduced fluorescence when multiple labeled nucleotides are incorporated.

More broadly, our results suggest that signal amplification via incorporation of multiple labeled nucleotides is possible with further sequence optimization followed by experimental validation. As incorporation of multiple labeled nucleotides with short spacers results in a limited increase in fluorescence, mitigating the diminishing signal increase with each incorporated labeled nucleotide will be essential for achieving signal amplification and/or accurate quantification of repeat bases. Achieving our optimum observed spacing of 11 nucleotides on a strand that could incorporate five labeled nucleotides would require a strand length of approximately 70 bases. We predict that such a sequence would result in over threefold signal amplification. In the case of DNA-based antibody readouts, longer strands could be a limiting factor since 80 base long DNA strands have been shown to substantially reduce antibody binding when compared to 30 base long strands.^36^ Further evaluation of the trade-off between antibody binding kinetics and the strand length will be necessary when developing readouts for antibody-based systems. Overall, the advantages of signal amplification incorporated into an *in situ* sequencing readout would be an improved assay timescale and fewer steps that introduce error or variability. However, it is possible that the maximum amount of signal amplification could be limited by self-quenching and/or truncation during elongation. Therefore, further investigation of this strategy as a strategy for signal amplification is warranted.

## III. CONCLUSIONS

In this study, we found that a polymerase elongation reaction in a polyacrylamide hydrogel is efficient when run to completion but reaches completion 1–2 orders of magnitude slower than predicted based on simple reaction or transport limitations. The minimal effect on reaction completion observed when quadrupling the concentration of the rate-limiting species, the polymerase, and an uneven fluorescence profile throughout the depth of the gel support our hypothesis that the process is transport limited. Additionally, the observed proportionality between the immobilized DNA concentration and the time for complete reaction throughout the gel supports the hypothesis of retarded diffusion of the polymerase due to interactions between the polymerase and the immobilized DNA. The broader implication for assay design is that consideration of the increased time for complete transport to ensure a complete, spatially uniform reaction throughout the gel is essential. Our work provides a jumping off point for the study of these phenomena toward the goal of more informed hydrogel-based assay design.

In this study, we also evaluated DNA sequence designs for the incorporation of multiple labeled nucleotides per strand via polymerase elongation. Our findings indicate that incorporating multiple labeled nucleotides per strand results in an increase in fluorescence. However, the increase tapers off with additional labeled nucleotides if the labeled nucleotides are spaced close together, such that only a 2.3-fold increase in fluorescence was observed when increasing the number of incorporated labeled nucleotides from 1 to 4. Increasing the spacing between labeled nucleotides from three unlabeled nucleotides to 11 labeled nucleotides increased the fluorescence signal by 18%, supporting our hypothesis of self-quenching. However, a crucial caveat that we discovered is that the final incorporated labeled nucleotide must not be too close to the end of the DNA strand; otherwise, a truncation product will form due to the inability of the polymerase to completely incorporate the nucleotide. The broader implication of this work is that careful DNA sequence design that increases the spacing between incorporated labeled nucleotides has the potential to improve the readout of repeated bases and introduce signal amplification into these readouts.

## IV. METHODS

### A. Transport and reaction models

For our transport model shown in Fig. 2, the partial differential equation with respect to time and one spatial dimension was solved using the pdpe numerical solver in MATLAB. The z-directional in-gel diffusion profile and Da plots were generated in MATLAB. All values used in the model are tabulated in Table S2.

For estimation of the retarded diffusion parameters, the 10 *μ*M FWHM data were fitted to the mean square displacement diffusion model in MATLAB using the lsqcurvefit function and an effective diffusion coefficient was extracted. A 95% confidence interval for the fitted diffusion coefficient was then obtained with the nlparci function.

### B. Chemical reagents

All DNA oligomers were purchased from Integrated DNA Technologies (Coralville, IA, USA), including oligomers with 5′ acrydite groups for incorporation into the gel matrix (see Table S3 for all DNA sequences). dATP (R0141), dGTP (R0161), and dCTP (R0151) were purchased as 100 mM stock solutions from Thermo Fisher Scientific (Waltham, MA, USA). Alexa Fluor aha dUTP (A32763), SYBR Gold nucleic acid stain (S11494), and Triton X-100 (BP-151) were also purchased from Thermo Fisher. Tetramethylethylenediamine (TEMED, T9281), ammonium persulfate (APS, A3678), 3-(trimethoxylsilyl)propyl methacrylate (440159), dichlorodimethylsilane (440272), and 30%T/3.3%C acrylamide/bis-acrylamide (29:1) (A3574) were purchased from Sigma-Aldrich (St. Louis, MO, USA). N-[3-[(3-Benzoylphenyl)formamido]propyl] methacrylamide (BPMAC) was custom synthesized by Pharm-Agra Laboratories (Brevard, NC, USA). De-ionized water (18.2 MΩ) was obtained using an Ultrapure water system from Millipore. TE and TBE buffers were prepared according to standard protocols.^37^

### C. In-solution polymerase experiments

DNA oligomers with complementary regions were mixed in a 1:1 ratio from 100 *μ*M stock solutions prepared in 1× TE buffer, and concentrated NaCl solution was added to reach a final concentration of 0.32 M NaCl. The mixture was then incubated for 20 min at 42 °C for hybridization to occur. Hybridized DNA was mixed with polymerase elongation solution to a final DNA concentration of 10 *μ*M in a total volume of 20 *μ*l and incubated for the specified times for the polymerase elongation reaction. Polymerase elongation solution consisted of 50 *μ*M each of dATP, dGTP, dCTP, Alexa Fluor 647 aha dUTP, and 0.125 U/*μ*l (unless otherwise specified) of Klenow (exo-) polymerase (NEB, Ipswich, MA, USA; M0212) in 10 mM Tris with 150 mM NaCl, 10 mM MgCl_2_, and 0.1% Triton X-100. After the polymerase elongation step, 0.2 *μ*g of each reaction was extracted, diluted to 15 *μ*l in 1× TBE buffer, mixed at 1:1 with 2× RNA loading dye (NEB; B0363S), and heated at 70 °C for 10 min to inactivate the polymerase and denature the DNA. The samples were cooled, and 20 *μ*l of sample was loaded into each lane of a 15% TBE-urea gel (Thermo Fisher Scientific). Gels were run at 200 V in 1× TBE buffer until the bromophenol blue band from the loading dye reached the bottom of the gel, ∼50 min. The run was then stopped, and the gels stained in 1× SYBR Gold for 30 min. Gels were imaged for SYBR Gold and Alexa Fluor 647 on an epifluorescence microscope (Olympus IX51 inverted fluorescence microscope equipped with a Photometrics CoolSNAP HQ2 CCD camera, an Applied Scientific Instrumentation motorized stage, and a Lumen Dynamics X-Cite shuttered mercury lamp light source, controlled by MetaMorph software by Molecular Devices) with a 2× objective (PlanApo by Olympus, N.A. = 0.08) using a 100 ms exposure time. Images were inverted in FIJI (ImageJ) software, and regions-of-interest (ROIs) were drawn around the bands to evaluate the fluorescence intensity. Background subtraction consisted of subtracting the fluorescence intensity of an ROI with the same size as the band that was placed in a blank section of the lane.

### D. Gel fabrication

Polyacrylamide gels were fabricated on silicon wafer substrates with 40 *μ*m rails patterned in SU-8, as previously reported.^4^ Gel precursor solutions containing acrylamide/bis-acrylamide stock solution (30%, 29:1) were prepared to a concentration of 8% T and 3.3% C. BPMAC was added to a concentration of 3 mM and DNA with a 5′ acrydite group added to a concentration of 10 *μ*M, unless otherwise specified, before degassing via sonication for 5 min. APS and TEMED were then added to the precursor to a concentration of 0.08% each before pipetting the solution between the SU-8 wafer (rendered hydrophobic by treatment with dichlorodimethylsilane) and a glass microscope slide functionalized with 3-(trimethoxysilyl)propyl methacrylate. After a 15-min chemical polymerization, the slides with the attached gels were lifted from the wafers and washed in 1× TBE buffer for at least 1 h.

### E. In-gel polymerase elongation experiments

Gels were fluidically addressed in a microarray cassette (1 × 16 hybridization cassette, Arrayit, Sunnyvale, CA, USA). In order to create a single-stranded overhang for polymerase elongation, solutions of DNA with complementary regions to the DNA immobilized in the gel were prepared to a concentration of 10 *μ*M in 0.32 M NaCl in 1× TE buffer and 100 *μ*l was pipetted into each region of the microarray cassette. The hybridization reaction was carried out at 21 °C for 2 h followed by aspirating off the DNA solution and washing twice with 100 *μ*l of 1× TBE buffer. Polymerase elongation solution (see the composition above) was then added (60 *μ*l per region of the microarray cassette), and the reaction was run at 21 °C for the specified times. The volume of reaction solution added to each gel region was over an order of magnitude greater than the gel volume to promote constant source diffusion of the reagents into the gel. After the elongation reaction, each region was briefly washed twice with 100 *μ*l of 1× TBE buffer before removing the gel from the cassette and washing for 30 min in a bath of wash buffer (10 mM Tris with 650 mM NaCl, 10 mM MgCl_2_, and 0.1% Triton X-100). Gels were then rinsed with de-ionized (DI) water and dehydrated under a N_2_ stream for imaging.

### F. Confocal microscopy

Following established methods from a previous study, confocal images were acquired on a Carl Zeiss LSM 710 AxioObserver inverted laser scanning confocal microscope using a C-Apochromat 40×/1.1 NA water-immersion objective with correction collar.^13^ The 633 nm line of a helium−neon laser was used to excite Alexa Fluor 647 and to perform reflected light confocal microscopy of the coverslip−sample interface to find the optimal correction collar setting. Fluorescence was imaged using a 488/561/633 nm dichroic filter and a pinhole set to 1.0 Airy units. Reflected light confocal images were acquired using a T80/R20 partial mirror over a field of view of 212.55 *μ*m × 212.55 *μ*m with cubic voxels of 0.71 *μ*m × 0.71 *μ*m× 0.10 *μ*m. The correction collar position was chosen using our previously established method.^13^

### G. Imaging and data processing

Imaging of the dehydrated gels was conducted using a GenePix 4300A microarray scanner equipped with a 635-nm laser. Imaging settings of 250 photomultiplier tube gain and 100% power were chosen, optimized for the maximum dynamic range without achieving saturation. Images were analyzed using FIJI (ImageJ) software. ROIs of 500 × 500 pixels were drawn in the center of the gel regions created by the microarray cassette and the integrated fluorescence, also known as area under the curve (AUC), evaluated. All fluorescence values were background subtracted by subtracting the AUC of a blank gel ROI of the same size.

### Ethics approval

No ethics approval is required for this work.

## SUPPLEMENTARY MATERIAL

See the supplementary material for additional experimental data and information.

## Data Availability

The data that support the findings of this study are available from the corresponding author upon reasonable request.
